# Superconductivity at 38 K at an electrochemical interface between an ionic liquid and FeSe_0.8_Te_0.2_ on various substrates

**DOI:** 10.1038/s41598-018-33121-7

**Published:** 2018-10-03

**Authors:** Shunsuke Kouno, Yohei Sato, Yumiko Katayama, Ataru Ichinose, Daisuke Asami, Fuyuki Nabeshima, Yoshinori Imai, Atsutaka Maeda, Kazunori Ueno

**Affiliations:** 1 Department of Basic Science, University of Tokyo, Meguro, Tokyo 153-8902 Japan; 2 Central Research Institute of Electric Power Industry, Yokosuka, Kanagawa 240-0196 Japan; 3 Department of Physics, Tohoku University, Sendai, 980-8578 Japan

## Abstract

Superconducting FeSe_0.8_Te_0.2_ thin films on SrTiO_3_, LaAlO_3_ and CaF_2_ substrates were electrochemically etched in an ionic liquid, DEME-TFSI, electrolyte with a gate bias of 5 V. Superconductivity at 38 K was observed on all substrates after the etching of films with a thickness greater than 30 nm, despite the different *T*_c_ values of 8 K, 12 K and 19 K observed before etching on SrTiO_3_, LaAlO_3_ and CaF_2_ substrates, respectively. *T*_c_ returned to its original value with the removal of the gate bias. The observation of *T*_c_ enhancement for these thick films indicates that the *T*_c_ enhancement is unrelated to any interfacial effects between the film and the substrate. The sheet resistance and Hall coefficient of the surface conducting layer were estimated from the gate bias dependence of the transport properties. The sheet resistances of the surface conducting layers of the films on LaAlO_3_ and CaF_2_ showed identical temperature dependence, and the Hall coefficient was found to be almost independent of temperature and to take values of −0.05 to −0.2 m^2^/C, corresponding to 4–17 electrons per FeSe_0.8_Te_0.2_ unit cell area in two dimensions. These common transport properties on various substrates suggest that the superconductivity at 38 K appears in the surface conducting layer as a result of an electrochemical reaction between the surface of the FeSe_0.8_Te_0.2_ thin film and the ionic liquid electrolyte.

## Introduction

FeSe is an iron-based superconductor with the simplest possible composition and exhibits superconductivity at 8.5 K^1^. FeSe has recently attracted considerable attention owing to the enhancements in the superconducting transition temperature (*T*_c_) that can be achieved through various methods. The *T*_c_ of a one-unit-cell FeSe thin film on a SrTiO_3_ substrate has been reported to take values of 105 K and 85 K based on an *in situ* resistivity measurement and a diamagnetic measurement, respectively^2,3^. Spectroscopic studies of monolayer and several-layer FeSe on SrTiO_3_ have revealed superconducting gaps corresponding to 65 K and 80 K by means of angle-resolved photoemission spectroscopy (ARPES) and scanning tunneling microscopy (STM), respectively^4–6^. These values are much higher than the bulk *T*_c_ values of all known iron-based superconductors. A *T*_c_ of 48 K has also been observed in several-layer FeSe on SrTiO_3_ with carrier doping by K ions^7^. This *T*_c_ enhancement has been suggested to originate from charge transfer from the oxide substrate to the ultrathin FeSe film^6^. *T*_c_ enhancements of up to approximately 40 K have also been reported following the insertion of cations or a (Li_0.8_Fe_0.2_)OH layer to the FeSe mother compound^8–14^. Recently, electrostatic carrier doping on ultrathin FeSe films and flakes has also been found to enhance *T*_c_ up to approximately 40 K^15–19^. The authors of these studies employed an electric double layer transistor (EDLT) configuration with an ionic liquid, diethylmethyl(2-methoxyethyl)ammonium bis(trifluoromethylsulfonyl)imide (DEME-TFSI), as a gate electrolyte for tuning the high-density carriers^20–22^. Since no additional phase other than FeSe was found in the X-ray diffraction (XRD) data^18^, the *T*_c_ enhancement was concluded to originate from electrostatic carrier doping. In addition, a thickness dependence study conducted by means of the electrochemical etching of FeSe showed that *T*_c_ enhancement occurred only when the film was thinner than 15 nm^16,19^, probably indicating that the interface between the film and the substrate also plays an important role in the *T*_c_ enhancement.

We have previously reported *T*_c_ enhancements in FeSe_1−x_Te_x_ thin films on various substrates^23–26^. The observed *T*_c_ values depend on both the Te content and the substrate material. For example, the *T*_c_ values for FeSe_0.8_Te_0.2_ films on CaF_2_ and LaAlO_3_ substrates are enhanced to 20 K and 12 K, respectively, in contrast to the *T*_c_ of 8 K observed for FeSe_0.8_Te_0.2_ on SrTiO_3_. This can be explained by differences in the a-axis lattice constants of FeSe_0.8_Te_0.2_ films on different substrates^24^. In this paper, we report the enhancement of *T*_c_ up to 38 K for thick FeSe_0.8_Te_0.2_ films on various substrates prepared via EDLT fabrication with the ionic liquid DEME-TFSI. By means of electrochemical etching with the ionic liquid^16^, the film thickness was varied. The application of a gate bias resulted in the formation of a surface conducting layer with a *T*_c_ of 38 K; with the removal of the gate bias, the surface conducting layer disappeared, causing *T*_c_ to return to its original value. We also estimated the transport properties of the surface conducting layer. The surface conducting layer exhibited electron conduction with a common dependence of the mobility on temperature on various substrates.

## Results

### Characterization of FeSe_0.8_Te_0.2_ thin films

**Figure 1:**
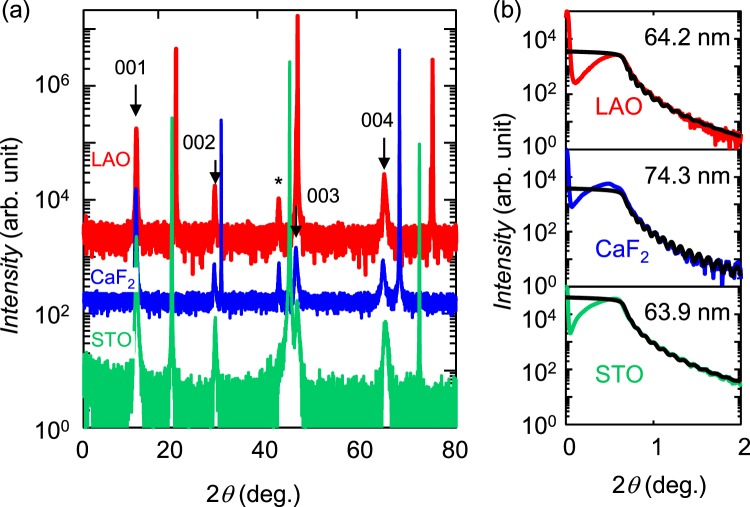
(**a**) X-ray diffraction (XRD) patterns of FeSe_0.8_Te_0.2_ thin films on LaAlO_3_ (LAO), CaF_2_ and SrTiO_3_ (STO) substrates. (**b**) X-ray reflectivity (XRR) patterns of the films. The black lines represent fit curves. The estimated thickness is shown in each panel.

The film thickness and crystal quality were examined via XRD measurements. Figure 1(a) shows the XRD patterns of FeSe_0.8_Te_0.2_ thin films fabricated on LaAlO_3_ (LAO), CaF_2_ and SrTiO_3_ (STO) substrates. All samples exhibited (001), (002) and (004) peaks, while the (003) peak for the film on the LAO substrate was obscured by a (002) peak of the substrate. The c-axis lattice constants for the films on the LAO, CaF_2_ and STO substrates were 5.69, 5.72 and 5.70 A, respectively, consistent with previous reports^23,24^. The full widths at half maximum (FWHMs) of the rocking curves for the (001) peak were 0.4, 0.7 and 1 deg. for the films on the LAO, CaF_2_ and STO substrates, respectively; these values are also almost the same as those reported previously (0.2–0.6 deg.)^23^, demonstrating that all of these films consisted of high-quality single-phase samples. X-ray reflectivity (XRR) measurements revealed clear thickness fringes for all films, as shown in Fig. 1(b), indicating a smooth surface and a sharp interface between the film and the substrate. In addition, all XRR curves were well fitted by the model structure, and we estimated the thicknesses as shown in Fig. 1(b). The film thicknesses were also confirmed by atomic force microscopy (AFM) images of the films.

### Superconducting properties of pristine and etched FeSe_0.8_Te_0.2_ thin films

**Figure 2:**
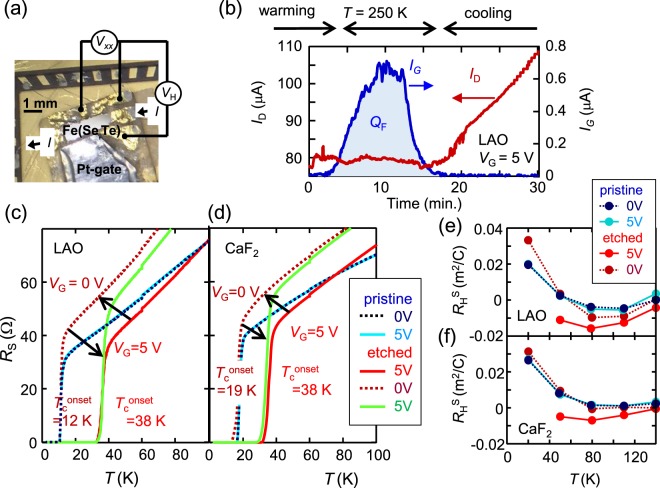
Changes in the superconducting and transport properties of FeSe_0.8_Te_0.2_ samples subjected to electrochemical etching and gating. (**a**) A photographic image of the electric double layer transistor (EDLT) device on the FeSe_0.8_Te_0.2_/LAO sample. The four-terminal resistance and the Hall resistance were simultaneously measured with the Hall bar electrodes. The ionic liquid electrolyte was placed between the film and the Pt gate. (**b**) The time dependences of the drain current (*I*_D_) and the gate current (*I*_G_) during electrochemical etching at *V*_G_ = 5 V and 250 K for the FeSe_0.8_Te_0.2_/LAO sample. *I*_G_ corresponds to the Faradaic current, and the product of *I*_G_ and time corresponds to the Faradaic charge (*Q*_F_). (**c**,**d**) The temperature (*T*) dependences of the sheet resistance (*R*_S_) for the FeSe_0.8_Te_0.2_ samples fabricated on the LAO and CaF_2_ substrates, respectively. Each panel shows the *R*_S_-*T* curves with and without gating for the pristine and etched samples. For each etched sample, bias voltages *V*_G_ of 5 V (red solid line), 0 V (red broken line), and 5 V (purple solid line) were applied in that order. For each pristine sample, bias voltages *V*_G_ of 0 V (blue broken line) and 5 V (blue solid line) were applied in that order. (**e**,**f**) The *T* dependences of the Hall coefficient (*R*_H_) for the samples on the LAO and CaF_2_ substrates, respectively. Each panel shows the *R*_H_-*T* curves with and without gating for the pristine and etched samples.

The superconducting properties of the samples when subjected to gating and etching were examined for a gate bias (*V*_G_) of 5 V. As shown in Fig. 2(a), the EDLT samples were patterned in Hall bars with six electrodes, and a Pt film was placed alongside each Hall-bar-instrumented sample to act as a gate electrode. Figure 2(c,d) show the temperature (*T*) dependences of the sheet resistance (*R*_S_) for samples of FeSe_0.8_Te_0.2_ films on LAO and CaF_2_ substrates (LAO and CaF_2_ samples), respectively. First, the *R*_S_*-T* curves of the pristine sample before etching were measured for *V*_G_ values of of 0 V and 5 V. Then, the temperature was increased to 250 K with a *V*_G_ of 5 V while monitoring the gate current (*I*_G_), as shown in Fig. 2(b). The channel was electrochemically etched, and the drain current (*I*_D_) was gradually decreased. The product of *I*_G_ and time is a Faradaic charge (*Q*_F_) that is proportional to the amount of charge of the reacted ions. After etching, *T* was decreased while maintaining *V*_G_ = 5 V, and the *R*_S_–*T* curve was measured. After several cycles of etching, the *V*_G_ dependence of the *R*_S_–*T* curve for the etched sample was measured via the following procedure: First, the *R*_S_–*T* curve was measured for a *V*_G_ of 5 V. Then, the temperature was increased to 250 K without *V*_G_, and the *R*_S_–*T* curve for *V*_G_ = 0 V was measured. Finally, a *V*_G_ of 5 V was again applied at 250 K, and the *R*_S_–*T* curve for *V*_G_ = 5 V was measured again. We show the *R-T* curves of the pristine and etched samples for *V*_G_ = 5 V, *V*_G_ = 0 V, and *V*_G_ = 5 V. The *R*_S_–*T* curves of the pristine samples remained almost unchanged between the *V*_G_ values of 0 V and 5 V. In contrast, *T*_c_ was enhanced after several cycles of etching at *V*_G_ = 5 V. *T*_c_ increased from 12 K to 38 K for the LAO sample and from 19 K to 38 K for the CaF_2_ sample. As shown in S. Fig. 3 in the Supplementary Information, critical magnetic field at 0 K was also enhanced from 47 T to 67 T on films on the LAO substrate. The coherence length at 0 K was 3.8 nm to 3.1 nm. With the removal of *V*_G_, *R*_S_ increased, and *T*_c_ returned to the value of the pristine sample. With the application of a *V*_G_ of 5 V, *R*_S_ slightly decreased, and *T*_c_ was enhanced to 38 K for both samples. Notably, the *R*_S_ value at *V*_G_ = 5 V after the removal of the initial *V*_G_ was larger than that before the removal of *V*_G_. Since the sheet resistance due to electrostatic carrier doping should always have the same value at *V*_G_ = 5 V, this difference suggests that the change in *R*_S_ can be attributed to some other origin than electrostatic carrier doping. In addition, the *T*_c_ for *V*_G_ = 0 V was different for the LAO and CaF_2_ sample because of different lattice constant of the FeSe_0.8_Te_0.2_ films. If the surface conducting layer is produced by an electrostatic carrier doping, it is natural that the surface conducting layer also show different *T*_c_ values for *V*_G_ = 5 V. Then, the *T*_c_ enhancement to 38 K for both samples also suggests that the origin of the carrier doping is not the electrostatic doping. This will be discussed later. Figure 2(e,f) show the *T* dependences of the Hall coefficient (*R*_H_) for the same samples shown in Fig. 2(c,d), respectively. *R*_H_ was almost zero above 80 K and showed an increase with decreasing temperature below 40 K for the pristine samples. In contrast, *R*_H_ was always negative at all temperatures for the etched samples at *V*_G_ = 5 V. The *R*_H_-*T* behavior returned to almost the original one after the application of *V*_G_ = 0 V. These results indicate that the etching at *V*_G_ = 5 V resulted in the formation of a conducting layer on the surface, with a *T*_c_ of 38 K, for both the LAO and CaF_2_ samples. Since *R*_H_ was negative, electron conduction dominated in the conductive layer. In addition, since the transport properties of the pristine and etched samples were almost the same for a *V*_G_ of 0 V, it can be concluded that the surface conductive layer disappeared with the removal of *V*_G_ from the etched sample.

### Thickness dependence of the superconducting properties of FeSe_0.8_Te_0.2_ thin films

**Figure 3:**
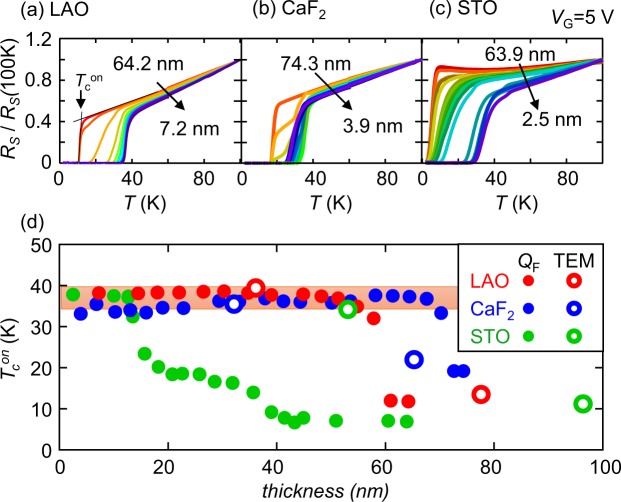
Variations in the superconducting transition temperature with changes in thickness for FeSe_0.8_Te_0.2_ samples on various substrates. (**a**–**c**) The *T* dependences of *R*_S_ (normalized to the *R*_S_ value at 100 K, *R*_S_(100 K)) for LAO, CaF_2_ and STO samples, respectively, subjected to electrochemical etching at *V*_G_ = 5 V. (**d**) The thickness dependences of *T*_c_^onset^ for the three samples. Filled symbols correspond to the data shown in (**a**–**c**), for which the thickness was estimated from *Q*_F_. Open symbols correspond to other samples whose thicknesses after etching were directly measured via TEM, as shown in Fig. 4.

The thickness dependence of the superconducting properties was also examined for the LAO, CaF_2_ and STO samples. Figure 3(a–c) show the *R*_S_-*T* curves of the LAO, CaF_2_ and STO samples, respectively, with various thicknesses (numbers of etching cycles). *R*_S_ is normalized to the *R*_S_ value at 100 K. For the LAO and CaF_2_ samples, *T*_c_ was enhanced after several cycles of etching. The CaF_2_ sample showed a two-step superconducting transition during the initial stage of etching. A similar two-step transition has been reported for an EDLT configuration on an FeSe flake with a gate bias of approximately 4 V and has been ascribed to the inhomogeneity of the carrier distribution^15^. In addition, the normalized *R*_S_-*T* curves after the *T*_c_ enhancement were nearly identical. In contrast, for the STO sample, *T*_c_ was gradually enhanced from 8 K to 38 K over many cycles. We estimated the onset temperature (*T*_c_^onset^) of superconductivity from the *R*_S_-*T* curve as shown in Fig. 3(a). We also estimated the thickness after *n* cycles of etching, *thickness(n)*, from the following equation:

$$thickness(n)=thickness(XRR)\times \sum _{i=1}^{n}{Q}_{F}/\sum _{i=1}^{n{\rm{\_}}total}{Q}_{F},$$

where *Q*_F_ is the Faradaic charge for each cycle, *thickness(XRR)* is the film thickness before etching as estimated from the XRR measurement, and *n*_total_ is the total number of cycles needed for the etching of the entire film. After the total etching of the film, the sample resistance is larger than MOhm. In addition, the film totally disappeared after the etching experiment. Therefore, we assumed the entire film was etched during the *n*_total_ cycles of etching. Figure 3(d) shows the thickness dependence of *T*_c_^onset^ for the LAO, CaF_2_ and STO samples. *T*_c_ started to increase only after two cycles of etching and saturated at 38 K after four cycles for the LAO and CaF_2_ samples. As reported in the previous paragraph, *T*_c_ decreased to its original value with the removal of *V*_G_ at 250 K, but it returned to 38 K after the next application of *V*_G_ = 5 V for the next cycle. The STO sample showed *T*_c_ enhancement from 8 K to 16 K at a thickness of 30 nm and a further *T*_c_ enhancement to 38 K at a thickness of 10 nm. Since *T*_c_ enhancement was observed for thick samples on all substrates, and since *T*_c_ changed with the application and removal of *V*_G_, we conclude that this *T*_c_ enhancement was not affected by the interface between the substrate and the film but instead originated from the surface conducting layer produced by *V*_G_ = 5 V.

**Figure 4:**
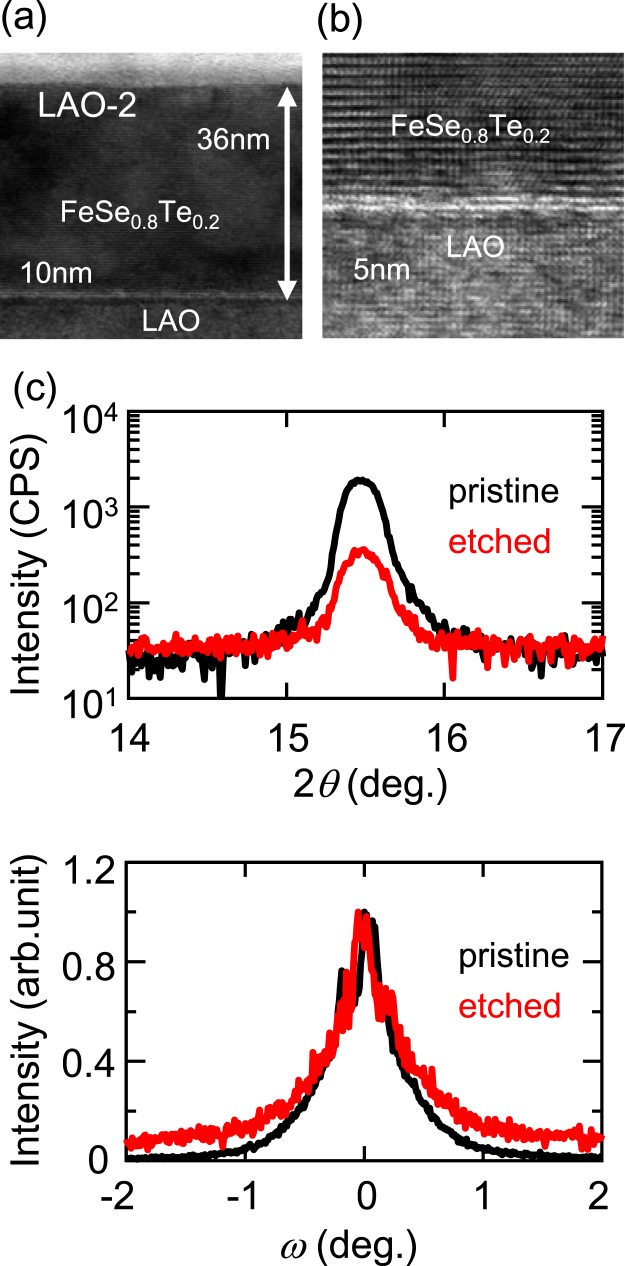
TEM and XRD data for the same sample on a LAO substrate before (pristine) and after (etched) electrochemical etching. (**a**,**b**) TEM images of the etched sample. (**c**,**d**) XRD patterns and rocking curves, respectively, for the (001) diffraction peak of the FeSe_0.8_Te_0.2_ film. The intensity is normalized to the intensity at *ω* = 0.

The thickness of the samples which showed the *T*_c_ enhancement was confirmed by means of transmission electron microscopy (TEM) and XRD measurements. We performed corresponding etching experiments using other samples on LAO, CaF_2_ and STO substrates and terminated the etching process after several cycles. All of the etched samples showed superconductivity at *T*_c_ values above 34 K. The film thickness after etching was directly obtained via TEM measurements. The thickness dependences of *T*_c_^onset^ for these samples are shown in Fig. 3(d). The FeSe_0.8_Te_0.2_ films on LAO, CaF_2_ and STO substrates all showed *T*_c_ enhancement for films with thicknesses greater than 30 nm. As shown in Fig. 4(a,b) and in S. Fig. 1(a,e) in the Supplementary Information, the interface between the substrate and the film was smooth for all films, and a clear periodicity of the atomic arrays was observed. The bright region at the interface probably indicates Se diffusion from the film into the substrate^27,28^. For the film on the LAO substrate, the clear periodicity remained at the surface, and no additional layer was found on the film, as shown in Fig. 4(a). On the CaF_2_ and STO substrates, a disordered FeO_x_ layer was found on the ordered region with clear periodicity. The *T*_c_ enhancement probably occurred in this ordered region. XRD patterns recorded before and after etching indicated that the peak position remained unchanged and that no new peak was present after etching, as shown in Fig. 4(c); the only observed difference was a decrease in the peak intensity. In addition, the FWHM of the rocking curve for the (001) peak remained unchanged, as shown in Fig. 4(d). These XRD data indicated that no new phase was created in the film by the etching process. Thus, no electrochemical reaction occurred in the bulk of the film; instead, such reactions took place only at the surface.

We also examined the thickness dependence of *T*_c_ for FeSe films on LAO and STO substrates. As shown in S. Fig. 2(a,b), these films showed *T*_c_ enhancements of up to 30 or 40 K upon etching. On STO substrates, only films with thicknesses below 12 nm showed *T*_c_ enhancement. This finding coincides with those of previous reports^16^. In contrast, a film with a thickness of 30 nm on the LAO substrate showed *T*_c_ enhancement, similar to the behavior of FeSe_0.8_Te_0.2_ films. Notably, several FeSe_0.8_Te_0.2_ samples on CaF_2_ and STO substrates showed *T*_c_ enhancement to above 37 K only for thicknesses below 10 nm. The different critical thicknesses for FeSe and FeSe_0.8_Te_0.2_ films on different substrates were probably due to differences in the homogeneity of the films. As shown in S. Fig. 1(a,e) in the Supplementary Information, a disordered region was observed in the FeSe_0.8_Te_0.2_ film on STO. In addition, a disordered Fe (or FeO_x_) layer was observed on top of the FeSe_0.8_Te_0.2_ films on CaF_2_ and STO substrates. These TEM images indicate that the film quality depends on the substrate and that the best quality is achieved for FeSe_0.8_Te_0.2_ films on LAO substrates. We consider that good film quality throughout the entire film is necessary for the occurrence of *T*_c_ enhancement for a thick film. Thus, although we did not perform TEM measurements of all of these samples, the lack of *T*_c_ enhancement to 38 K for the thick films on some samples might have been due to insufficient film homogeneity, especially near the surface.

## Discussion

Finally, we discuss the origin of the *T*_c_ enhancement. The *T*_c_ enhancement has been reported to be due to charge accumulation on the surface of the FeSe^15,16,18^. However, an electrochemically reacted layer on the surface may also show a high *T*_c_, since FeSe samples intercalated with alkali ions and/or organic molecules present *T*_c_ values above 40 K^8,9^. To distinguish electrostatic charge accumulation from electrochemical reaction, we estimated the sheet resistance and Hall coefficient of the surface layer. The resistance tensor, *ρ*, and the conductance tensor, *σ*, are represented by the following equations:

1 $$\begin{array}{c}\rho =(\begin{array}{cc}{R}_{S} & -{R}_{H}B\\ {R}_{H}B & {R}_{S}\end{array})\end{array}$$

2 $$\begin{array}{c}\sigma ={\rho }^{-1}=\frac{1}{{R}_{S}^{2}+{R}_{H}^{2}{B}^{2}}(\begin{array}{cc}{R}_{S} & {R}_{H}B\\ -{R}_{H}B & {R}_{S}\end{array})\sim (\begin{array}{cc}\frac{1}{{R}_{S}} & \frac{{R}_{H}B}{{R}_{S}}\\ -\frac{{R}_{H}B}{{R}_{S}} & \frac{1}{{R}_{S}}\end{array})\end{array}$$

where *B* is the magnetic field applied during the Hall measurement. The *σ* of a sample at *V*_G_ = 5 V is equal to the sum of the *σ* values of the sample at *V*_G_ = 0 V and of the surface conducting layer produced by a *V*_G_ of 5 V. Therefore, the sheet resistance and Hall coefficient of the surface conducting layer, *R*_S_^surface^ and *R*_H_^surface^, obey the following equations:

3 $$\begin{array}{c}\frac{1}{{R}_{xx}({V}_{G}=5V)}=\frac{1}{{R}_{xx}({V}_{G}=0V)}+\frac{1}{{R}_{xx}^{surface}}\end{array}$$

4 $$\begin{array}{c}\frac{{R}_{H}({V}_{G}=5V)}{{R}_{xx}({V}_{G}=5V)}=\frac{{R}_{H}({V}_{G}=0V)}{{R}_{xx}({V}_{G}=0V)}+\frac{{R}_{H}^{surface}}{{R}_{xx}^{surface}}\end{array}$$

**Figure 5:**
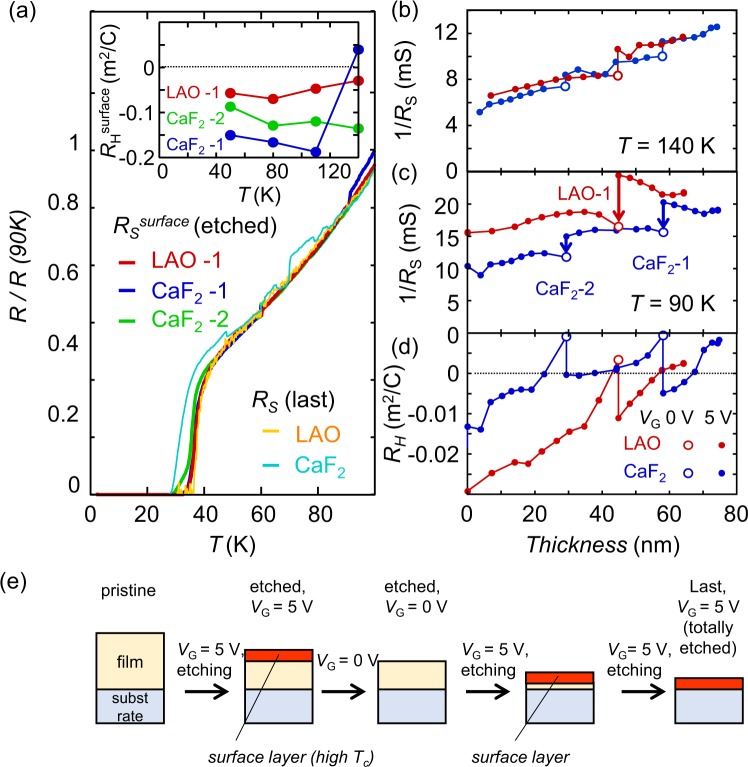
Superconducting transition for the surface conducting layer and changes in the transport properties with electrochemical etching for various samples. (**a**) The *T* dependence of the *R*_S_ values of the surface conducting layer (*R*_S_^surface^), normalized to the value at 90 K. *R*_S_^surface^ was estimated from the change in the sheet conductance between *V*_G_ values of 5 V and 0 V. Data for three *V*_G_ cycles of two samples, labeled as LAO-1, CaF_2_-1 and CaF_2_-2 in (**c**), are shown. The *R*_S_-T curves just before the last etching cycle for the LAO and CaF_2_ samples are also plotted. The inset shows the *R*_H_ values of the surface conducting layers of the LAO-1, CaF_2_-1 and CaF_2_-2 samples as a function of temperature. (**b**–**d**) Values of 1/*R*_S_ at 140 K and 50 K and of *R*_H_ at 50 K, respectively, as functions of the film thickness for the LAO and CaF_2_ samples. *V*_G_ was changed from 5 V to 0 V once and twice during the etching of the LAO and CaF_2_ samples, respectively. (**e**) Schematic illustration of the evolution of a sample during etching. A surface layer is formed during etching and disappears with the removal of the gate bias.

As shown in Fig. 5(b–d), we examined the changes in the sheet resistance and Hall coefficient for one *V*_G_ cycle of a LAO sample (LAO-1, as shown in Fig. 2(c)) and two *V*_G_ cycles of a CaF_2_ sample (CaF_2_-1 and CaF_2_-2, where the data for CaF_2_-1 are shown in Fig. 2(d)). Figure 5(a) shows the temperature dependence of *R*_S_^surface^ normalized to the value at 90 K. The *R*_S_*-T* curves for the LAO and CaF_2_ samples just before the last etching cycle, *R*_S_(last), are also plotted. All curves follow almost the same profile. This indicates that the transport properties, such as the electron mobility and scattering time, of all samples exhibited identical temperature dependences. As shown in the inset of Fig. 5(a), *R*_H_ was almost independent of temperature and negative for all samples. Electron-type conduction is a common feature in previous reports on the *T*_c_ enhancement of FeSe^7,15–18^, and the vanishing of the hole pocket at the Fermi level has been considered to be the origin of the *T*_c_ enhancement^7,15,16^. The observed Hall coefficient values of 0.05 to 0.2 m^2^/C correspond to 4–17 electrons per unit cell in two dimensions (0.376 nm × 0.376 nm). If such a high density of carriers were electrostatically accumulated on the surface, then electrons would be strongly scattered at the surface, and the scattering time should change with the variation in the accumulated carrier density^29^. However, no such change was observed in the *R-T* curves. In addition, as shown previously in Fig. 2(c,d), the removal of *V*_G_ irreversibly changed *R*_S_, suggesting that the origin of the change in *R*_S_ is some other phenomenon than electrostatic carrier doping. Therefore, a different cause of carrier doping other than the electric field effect is likely responsible for the *T*_c_ enhancement.

Both the irreversible change in *R*_S_ with *V*_G_ and the lack of variation in the electron mobility with the carrier doping can be explained by assuming that the surface conducting layer is formed not by the accumulation of electrostatic charge but by an electrochemical reaction between the FeSe_0.8_Te_0.2_ and the ionic liquid. We hypothesize that the etching of the film and the formation of the surface conducting layer occurred simultaneously with the application of the *V*_G_ of 5 V. One potential candidate of the forming reaction of the surface conducting layer is an electrochemical intercalation of DEME^+^ ion,

$${{\rm{FeSe}}}_{{\rm{0.8}}}{{\rm{Te}}}_{{\rm{0.2}}}+{{\rm{DEME}}}^{+}{+{\rm{e}}}^{-}+\to {{\rm{FeSe}}}_{{\rm{0.8}}}{{\rm{Te}}}_{{\rm{0.2}}}({\rm{DEME}}).$$

Since observed *Q*_F_ during the reaction was much larger than *Q*_F_ needed for this reaction, other electrochemical reaction, such as electrochemical decomposition of DEME-TFSI and dissolution of FeSe_0.8_Te_0.2_, also occurs. We discussed on the possible electrochemical reactions in the Supplementary Information. In addition, we hypothesize that when this *V*_G_ was removed, the surface conducting layer disappeared, probably due to decomposition or peeling off from the surface. Then, the abrupt decrease in the sheet conductance occurred with the removal of *V*_G_. In addition, both the electron mobility and the volume charge carrier density should be identical among LAO-1, CaF_2_-1 and CaF_2_-2. This hypothesis was also supported by the change in the sheet conductance with the repeated etching of the LAO and CaF_2_ samples, as shown in Fig. 5(d). The sheet conductance at 50 K decreased with the removal of *V*_G_ and, with repeated etching, gradually increased after this reduction. This behavior can be explained by an increase in the thickness of the surface conducting layer with repeated etching. If the conductance of the surface conducting layer is higher than that of the bulk FeSe_0.8_Te_0.2_ film at 50 K, then repeated etching will increase the sheet conductance at low temperatures. As the number of etching cycles increases, the ratio of the thickness of the surface conducting layer to the total film thickness will increase. Then, just before the film is totally removed, the surface conducting layer will cover almost the entire film. Consistent with this picture, the temperature dependences of the sheet resistance just before the last etching cycle for both the LAO and CaF_2_ samples were also identical to that for the surface conducting layer, as shown in Fig. 5(a). We also examined two dimensionality of the superconductivity on the surface conducting layer. When the superconducting layer is thinner than the superconducting coherence length, it behaves as a two dimensional superconductor. However, as shown in S. Fig. 4 in the Supplementary Information, the surface conducting layer did not behave as a two dimensional superconductor. This suggests that superconducting layer is electrochemically formed and relatively thick.

## Conclusion

In conclusion, a surface conducting layer with a *T*_c_ of 38 K was formed with the electrochemical etching of FeSe_0.8_Te_0.2_ thin films on LAO, CaF_2_ and STO substrates. Since the thicknesses of all etched samples with *T*_c_ values of 38 K were greater than 30 nm, the enhancement of *T*_c_ cannot be related to any interaction between the film and the substrate. In addition, *T*_c_ enhancement was also observed for an FeSe thin film on a LAO substrate with a thickness of approximately 30 nm. The surface conducting layer again showed almost identical temperature dependences between the sheet resistance and the Hall coefficient. This finding suggests that the surface conducting layer is formed not by the accumulation of electrostatic charge on the FeSe_0.8_Te_0.2_ surface but by an electrochemical reaction between the FeSe_0.8_Te_0.2_ and the ionic liquid electrolyte. Hall coefficient measurements showed that the surface conducting layer contained 4–17 electrons per unit cell in two dimensions, with an overall negative charge. From TEM measurements, we could observe a smooth interface between the substrate and the film and a clear periodicity of the atomic arrays in the etched FeSe_0.8_Te_0.2_ film on the LAO substrate. These observations indicate that the formation of the surface conducting layer did not affect the bulk region of the film, and the surface conducting layer completely disappeared with the removal of *V*_G_. We consider that previous studies on carrier doping in ultrathin FeSe film can be classified into two groups: those that show a *T*_c_ of approximately 40 K for a several-layer FeSe film and those that show a *T*_c_ above 65 K for a monolayer FeSe on STO. Our results indicate that the electrochemical doping of FeSe and FeSe_0.8_Te_0.2_ can result in the formation of a superconducting layer with a *T*_c_ of approximately 40 K and that no interfacial interaction is necessary for the enhancement of *T*_c_ to 40 K. However, we think that the interface between FeSe and STO is probably essential for the enhancement of *T*_c_ above 65 K. We believe that it will be possible to prepare monolayer FeSe with a *T*_c_ above 65 K with further study of the electrochemical etching of FeSe.

## Methods

FeSe and FeSe_0.8_Te_0.2_ thin films were deposited by means of pulsed laser deposition (PLD) with a KrF eximer laser and an FeSe or FeSe_0.8_Te_0.2_ polycrystalline target. The fabrication conditions are described in detail elsewhere^30,31^. We used a commercially available STO (001) substrate with a step-and-terrace surface, a LAO (001) substrate and a CaF_2_ (001) substrate. AFM and XRD measurements were carried out prior to device fabrication. Au(100 nm)/Ti (20 nm) films were formed via electron-beam evaporation at a base pressure of 10^−5^ Torr. Since FeSe_0.8_Te_0.2_ thin films can be damaged by exposure to high temperatures (above 100 deg. Celsius) and water during the standard processes of photolithography and dry etching, we employed a sandblasting technique at room temperature for the fabrication of Hall bar electrodes. The films were coated with a dry film resist patterned via photolithography and were etched by sandblasting with alumina emery (#220). The Hall bar electrodes and wires were coated with a silicone sealant to prevent electrochemical reactions between the electrolyte and the Au/Ti electrode. The ionic liquid DEME-TFSI was dropped onto the channel area of the Hall bar configuration and the Pt film. Electrochemical etching and transport measurements were carried out in a He atmosphere with a Quantum Design Physical Property Measurement System (PPMS) at temperatures from 300 K to 2 K.

## Electronic supplementary material

Supplementary Information
