# Supplementary material for: Superconductivity at 38 K at an electrochemical interface between an ionic liquid and FeSe0.8Te0.2 on various substrates
Source: Sci Rep. 2018 Oct 3;8:14731. doi: 10.1038/s41598-018-33121-7 (PMC6170459; doi:10.1038/s41598-018-33121-7)
Supplement: Supplementary file 1 — Supplementary Information [file 41598_2018_33121_MOESM1_ESM.docx]

**Supplementary Information**

**Superconductivity at 38 K in an electrochemical interface between ionic liquid and FeSe_0.8_Te_0.2_ on various substrates**

Shunsuke Kouno,^1^ Yohei Sato,^1^ Yumiko Katayama,^1^ Ataru Ichinose,^2^ Daisuke Asami,_1_ Fuyuki Nabeshima,^1^ Yoshinori Imai,^3^ Atsutaka Maeda,^1^ and Kazunori Ueno^1^*

*^1^Department of Basic Science, University of Tokyo, Meguro, Tokyo 115-8902, Japan*

*^2^Central Research Institute of Electric Power Industry, Yokosuka, Kanagawa 240-0196, Japan*

*^3^Department of Physics, Tohoku University, Sendai 980-8578, Japan*

**TEM images after etching for samples on CaF_2_ and SrTiO_3_**

Scanning TEM experiments were performed on FeSe_0.8_Te_0.2_ films on CaF_2_ and STO substrates. *T*_c_ was changed from 22.0 K to 35.5 K for the film on the CaF_2_ substrate and 11.2 K to 34.K for the film on the STO substrate after several cycles of the etching. Topographic images for both films include a bright area with a thickness of several atomic layers at the interface between the substrate and the film, probably indicating Se diffusion from the film into the substrate. In addition, both films include a bright, disordered area on the top of a dark area with a clear periodic atomic arrays. Since O and Fe atoms are observed but Se atoms are not observed in the disordered area, this area corresponds to the oxidized Fe (FeO_x_) layer. In addition, there are no FeO_x_ peaks from the 2*θ*-*θ* measurement by the XRD before and after the etching, the FeO_x_ is a polycrystal or an amorphous. The FeO_x_ layer possibly originates from chemical reaction between air and a residual Fe layer after the electrochemical etching of FeSe. We estimated a film thickness from the dark, well-crystallized FeSe area and plotted in Fig. 3 in the manuscript.

S. Figure 1 Topographic image of scanning TEM and EDX mapping images of Fe, Se and O. (a)-(d) correspond to FeSe_0.8_Te_0.2_ film on CaF_2_ substrate. (e)-(h) correspond to FeSe_0.8_Te_0.2_ film on SrTiO_3_ substrate.

**Thickness dependence of superconducting critical temperature for FeSe thin films on LAO and STO substrates**

One FeSe film and three FeSe films were fabricated on LAO and STO substrates, respectively. The films were etched for *V*_G_ of 5 V and the temperature dependence of the resistance was measured after the each etching cycle. Temperature dependence of the resistance is shown in S. Fig. 2(a) and (b). Critical temperature rapidly increased for the film on the LAO substrate. In contrast, the film on the STO substrate showed a *T*_c_ increase with a thickness of around 10 nm. S. Figure 2(c) shows the thickness dependence of *T*_c_^onset^ for all FeSe samples.

S. Figure 2 (a), (b) Temperature dependence of sheet resistance *R* normalized by *R* at 100 K with various thicknesses *t*. (c) Thickness dependence of superconducting critical temperature *T*_c_^onset^ for FeSe samples on LAO and STO substrates.

**Electrochemical reactions on the FeSe_0.8_Te_0.2_ / DEME-TFSI interface**

For the thickness estimation, we assumed that the Faradaic charge *Q*_F_ for each cycle is proportional to an etched thickness of the Fe(Se_0.8_Te_0.2_) film during the cycle. The observed *Q*_F_ is sum of *Q*_F_ by all electrochemical reactions in the ionic liquid. S. Table 1 shows *Q*_F_ during an etching process of the FeSe_0.8_Te_0.2_ films and corresponding numbers of electrons per etched Fe atoms. 11.2 – 21.4 electrons flowed during etching of one Fe atom. The difference of the electron count probably originates from *Q*_F_ by the side-reactions between the ionic liquid (or impurity in the ionic liquid) and electrodes. Since *Q*_F_ by the side-reactions would be changed by amount of impurities in the ionic liquid and coverage of ionic liquid on the electrodes, *Q*_F_ also showed the sample dependence.

Superconductivity has been reported on alkali-metal doped or (Li_0.8_Fe_0.2_)OH inserted FeSe with *T*_c_ of 40 K. Then, we think that cation intercalated Fe(Se,Te) is a potential candidate of the conducting layer. For example, if DEME^+^ ions in the ionic liquid are electrochemically inserted, following reaction would occur with *V*_G_ of 5 V.(cathode reaction)

FeSe_0.8_Te_0.2_ + DEME^+^ + e^-^ -> FeSe_0.8_Te_0.2_(DEME)

In addition, since water is a major impurity in the ionic liquid, insertion of H^+^ ions to (Se,Te) can also occur. When these reaction occurs, carrier density of the reacted layer increased by one electron/lattice.

However, an electrochemical etching of the FeSe_0.8_Te_0.2_ simultaneously occurs during the electrochemical doping. In addition, the observed electron count (>10 electrons) are much larger than electrons for the doping reaction, suggesting other electrochemical reaction. Since *V*_G_ of 5 V is around the electrochemical window of the ionic liquid, DEME-TFSI, electrochemical decomposition of DEME^+^ and/or TFSI^-^ probably occurs, resulting in a formation of chemically reactive species. Indeed, the color of the ionic liquid after applying *V*_G_=5V was changed to be yellowish. We think that such reactive species probably react with FeSe_0.8_Te_0.2_, resulting in the etching of the film.

In a water-based electrolyte, FeSe are electrochemically deposited by following raction.[S1]

FeSe + 4H_2_O -> Fe^2+^ + SeO_4_^2-^ + 8H^+^ + 8e^-^

Then, similar electrochemical decomposition of the surface conducting layer can occur by water in the ionic liquid for *V*_G_ = 0 V.(anode reaction)

FeSe_0.8_Te_0.2_(DEME) + 4H_2_O -> Fe^2+^ + Se(Te)O_4_^2-^ +DEME^+^ + 8H^+^ + 9e^-.^

Another possible reaction is dissolution of the surface conducting layer without oxidation of selenium,

FeSe_0.8_Te_0.2_(DEME) -> Fe^2+^ + Se(Te)^2-^ +DEME^+^ + e^-.^

Since the etching experiment was carried out at low temperature in vacuum, we cannot make detailed electrochemical measurements on this interface. For further understanding the results, investigations at room temperature with large FeSe0.8Te0.2 film electrode is needed.

| sample  (substrate) | Faradaic charge  $Q_{F} (\mathrm{mC})$ | Etched thickness  $A (nm)$ | Channel area  $S (\mathrm{mm}^{2})$ | Electrons per  one Fe atom |
| --- | --- | --- | --- | --- |
| sample 1(LAO) | 5.28 | 64.2 | 1.18 | 16.9 |
| sample 2 (CaF_2_) | 5.76 | 74.3 | 1.00 | 18.9 |
| sample 3 (STO) | 6.16 | 63.9 | 1.09 | 21.4 |
| sample 4 (LAO) | 1.76 | 27.4 | 1.08 | 14.4 |
| sample 5(CaF_2_) | 1.38 | 31.1 | 0.96 | 11.2 |

S. Table1.　Faradaic charge during an etching process of the FeSe_0.8_Te_0.2_ films and corresponding numbers of electrons per etched Fe atoms. Samples 1-3 correspond to the devices shown in Fig. 3, and samples 4,5 correspond to the samples for the TEM measurements.

**Superconducting properties of the FeSe_0.8_Te_0.2_ films on LaAlO_3_ substrate**

We examined critical magnetic field for a pristine FeSe_0.8_Te_0.2_/LAO film and the surface conducting layer with *V*_G_ =5 V (the sample is identical to the TEM sample shown in Fig. 4). As shown in S. Fig. 3, critical magnetic field of the surface conducting layer is higher than that of the pristine film.

S. Figure 3 Temperature dependence of the critical magnetic field for the pristine film and the surface conducting layer with *V*_G_ = 5V. The critical magnetic field and the coherence length at *T* = 0 K is shown in the right side.

When a thickness of a superconducting film is smaller than the superconducting coherence length *ξ*, it behaves as a two dimensional superconductor. In that case, Berezinskii-Kosterlitz-Thouless (BKT) transition occurs below *T*_c_. We analyzed the data in Fig. 2 (c) (FeSe_0.8_Te_0.2_/LAO with *V*_G_ of 0 V and 5 V) with the Halperin-Nelson formula. [S2]

$$R_{S}=R_{0}\exp\left( -2b\left( \frac{T}{T_{0}}-1 \right)^{\frac{1}{2}} \right)$$

This formula is changed to following equation,

$$\left( \frac{d\log\left( R_{S} \right)}{dT} \right)^{-2/3}=\left( \frac{b}{T_{0}} \right)^{-\frac{2}{3}}\left( \frac{T}{T_{0}}-1 \right)$$

When the superconductor is two dimensional, *T*_0_ is much smaller than the critical temperature *T*_c_, and *T*_0_ equal to the BKT transition temperature *T*_BKT_. As shown in S.Fig. 4(b), although *R-T* curve seemed to be fitted by the formula, the transition temperature *T*_0_ is almost identical to *T*_c_. In addition, as shown in S. Fig. 4 (a), the fitted curve follows onset of the superconducting transition. These indicate that our sample did not show the BKT transition, and suggest that the thickness of the superconducting layer is larger than the coherence length.

S. Figure4 Curve fitting by the Halperin-Nelson formula. (a) *R-T* curves the fitted curve for the FeSe_0.8_Te_0.2_ film on LaAlO_3_ substrate for *V*_G_ of 0 V and 5 V. (c) Linear fitting of $\left( \frac{d\log\left( R_{S} \right)}{dT} \right)^{-2/3}$ as a function of temperature. *T*_c_^mid^ and *T*_0_ are also shown.

**References**

[S1] S. Demura, H. Okazaki, T. Ozaki, H. Hara, Y. Kawasaki, K. Deguchi, T. Watanabe, S. J. Denholme, Y. Mizuguchi, T. Yamaguchi, H. Takeya, Y. Takano, Solid State Commun. 154, 40 (2013).

[S2] Halperin, B. I. & Nelson, D. R. , J. Low Temp. Phys. 36, 599-616 (1979).
